# Iodoprophylaxis and thyroid autoimmunity: an update

**DOI:** 10.1007/s12026-021-09192-6

**Published:** 2021-04-29

**Authors:** Claudia Teti, Marta Panciroli, Elena Nazzari, Giampaola Pesce, Stefano Mariotti, Antonella Olivieri, Marcello Bagnasco

**Affiliations:** 1 Endocrinology and Diabetology Unit, ASL 1, Imperia, Italy; 2 Department of Internal Medicine and Medical Specialties (DiMI), Genoa University, Viale Benedetto XV, 6 I-16132 Genova, Italy; 3 Department of Neurosciences, Rehabilitation, Ophthalmology, Genetics, Maternal and Child Health, University of Genoa, Genoa, Italy; 4 Endocrinology and Diabetology Unit, ASL 2, Savona, Italy; 5 Laboratory of Autoimmunology, IRCCS Ospedale Policlinico San Martino, Genova, Italy; 6 Department of Medicine and Public Health, University of Cagliari, Cagliari, Italy; 7 Department of Cardiovascular and Endocrine-Metabolic Diseases and Aging, Italian National Institute of Health, Viale Regina Elena, 299, 00161 Roma, Italy

**Keywords:** Thyroid, Thyroid autoimmunity, Iodine, Iodized salt, Goiter

## Abstract

Adequate iodine intake is necessary for normal thyroid function. Iodine deficiency is associated with serious complications, but also iodine excess can lead to thyroid dysfunction, and iodine supplementation aimed to prevent iodine deficiency disorders has been associated with development of thyroid autoimmunity. The epidemiology of thyroid diseases has undergone profound changes since the implementation of iodoprophylaxis, notably by means of iodine-enriched salt, specifically resulting in decreased prevalence of goiter and neonatal hypothyroidism, improved cognitive function development in infancy, and reduced incidence of more aggressive forms of thyroid cancer. The main question we address with this review is the clinical relevance of the possible effect on autoimmunity exerted by the use of iodine-enriched salt to correct iodine deficiency. In animal models, exogenous iodine is able to trigger or exacerbate thyroid autoimmunity, but it is still not clear whether the observed immunological changes are due to a direct effect of iodine on immune response, or whether they represent a secondary response to a toxic effect of iodine on thyroid tissue. Previous iodine status of a population seems to influence the functional thyroid response to increased iodine intake and possibly the development of thyroid autoimmunity. Moreover, the prevalence of thyroid antibodies, regarded as hallmark of autoimmune thyroid disease, varies between populations under the influence of genetic and environmental factors, and the presence of thyroid antibodies does not always coincide with the presence of thyroid disease or its future development. In addition, the incidence of autoimmune diseases shows a general increasing trend in the last decades. For all these reasons, available data are quite heterogeneous and difficult to analyze and compare. In conclusion, available data from long-term population surveys show that a higher than adequate population iodine intake due to a poorly controlled program of iodine prophylaxis could induce thyroid dysfunction, including thyroid autoimmunity mostly represented by euthyroid or subclinical hypothyroid autoimmune thyroiditis. Close monitoring iodine prophylaxis is therefore advised to ensure that effects of both iodine deficiency and iodine excess are avoided.

## Introduction: thyroid autoimmunity and iodine

Autoimmune thyroid diseases (AITD) are the most common autoimmune disorders [1, 2]. Autoimmune thyroiditis (AT) is the paradigm of “destructive” organ-specific autoimmunity, and Graves’ disease (GD) is the unique clinically relevant case of organ-specific autoimmune disease resulting in hyperfunction of the target organ and the most common cause of hyperthyroidism with the possible exception of areas with severe iodine deficiency. The multi-faceted clinical presentation is due to multiple pathogenetic mechanisms involving (cell-mediated in autoimmune hypothyroidism due to thyroid destruction and autoantibody-mediated in autoimmune hyperthyroidism) [3–5]. In any case, the presence of thyroid autoantibodies has been regarded for decades as a hallmark of AITD: notably, antibodies directed against thyroid peroxidase (TPOAb) or thyroglobulin (TgAb) [6]. Such autoantibodies are present in both AT and GD, although more frequently and at higher levels in the former. The prevalence of thyroid antibodies varies between populations under the influence of genetic [7–10] and environmental [7, 11] factors.

The prevalence of AT is far higher than that of GD (about 5- to tenfold) [1]. Therefore, the presence of such autoantibodies is predominantly associated with decreased thyroid function. It has to be underlined that the presence of circulating TPOAb or TgAb does not always coincide with the presence of disease: clinical disease requires significant organ damage, in which cell-mediated mechanisms play a major role, as above mentioned. Such thyroid autoantibodies can be predictive of disease development over time [12] but also be a transient phenomenon; thus, their prevalence is higher than clinical disease [13]. On the other hand, TSH-receptor autoantibodies (TRAb) are the only autoantibodies displaying a direct pathogenetic role, most often causing hyperthyroidism due to their thyroid-stimulating activity: in fact, since the development of highly sensitive assays [14], their prevalence is closely similar to that of clinical GD*.*

Normal thyroid activity is dependent on iodine intake. In fact, iodine is the most important environmental factor in upholding thyroid function [15].

Iodine deficiency has been since centuries a major epidemiological problem. In fact, since the very beginning of life outside the oceans, some 400 million years ago, the availability of iodine in the environment became very poor: there is circumstantial very early evidence for iodine deficiency disorders in Homo sapiens as well as in Neanderthals, and iodine availability and the ability to use it has probably been an important factor in human evolution [16]. Endemic goiter is the most visible sign of iodine deficiency, but its most devastating consequence is brain damage causing mental retardation in children.

In Table 1, the optimal levels of iodine intake by age and population group according to the World Health Organization (WHO) and the United States Institute of Medicine (IOM) are reported. Iodine supplementation through salt iodization, implemented by the WHO and the International Council for the Control of Iodine Deficiency Disorders (ICCIDD, now Iodine Global Network), is a worldwide, effective strategy for preventing iodine deficiency-related problems. Its safety and efficacy profile has been extensively investigated, and benefits far outweigh the potential iodine-induced risks [17, 18]. Iodine prophylaxis through iodized salt has been shown to modulate the pattern of thyroid diseases and also to exert a pivotal role in abating iodine deficiency disorders (IDD) [19, 20].

**Table 1:** Recommendations for iodine intake (µg/d) by age or population group

	IOM			WHO
Age or population group	EAR	AI or RDA	Age or population group	RNI
Infants 0–12 months		110–130	Children 0–5 yr	90
Children 1–8 yr	65	90	Children 6–12 yr	120
Children 9–13 yr	73	120		
Adults ≥ 14 yr	95	150	Adults ≥ 12 yr	150
Pregnancy	160	220	Pregnancy	250
Lactation	200	290	Lactation	250

*IOM*, United States Institute of Medicine; *EAR*, estimated average requirement; *AI*, adequate intake; *RDA*, recommended daily allowance; *RNI*, recommended nutrient intake; *WHO*, World Health Organization

Iodine excess also exerts important effects on thyroid function, being able to transiently block it (Wolff-Chaikoff effect), or producing long-lasting hypothyroidism; moreover, iodine excess may cause transient destructive thyrotoxicosis via toxic damage, or sustained hyperthyroidism in the presence of autonomously functioning tissue [21]. Finally, and more relevant to this review, thyroid autoimmunity can be driven by exposure to iodine excess.

For years, several mechanisms have been suggested to explain the immunomodulatory effects of iodine and specifically the relationship between the level of iodine intake and thyroid autoimmunity [22, 23]. It is still not clear whether the observed immunological changes are due to a direct iodine effect on immune effector cells, or whether they represent a secondary response to a metabolic and/or toxic effect of iodine on thyroid tissue. It has been hypothesized that a sudden shift from very low to high iodine intake may induce damage to thyroid tissue by free radicals [24, 25] whose importance in triggering/exacerbating thyroid autoimmunity is presently widely recognized [26–28].

The role of iodine in inducing thyroid autoimmunity is strongly supported by animal models. Excess of iodine can anticipate and exacerbate the occurrence of spontaneous thyroiditis in genetically predisposed animals, by increasing the immunogenicity of thyroglobulin (Tg) [23, 29–31]*.* Thyroglobulin is an iodinated protein and is an integral part of the hormone synthesizing mechanisms of the thyroid follicle.

Some authors described iodinated thyroglobulin eliciting a greater immune response compared with less iodinated thyroglobulin molecules in in vitro experiments [29, 32, 33]. It has been demonstrated that the enhanced iodination of thyroglobulin induces the expression of new cryptic epitopes on the molecule that could be responsible for triggering the autoimmune process [30]. The rise of TgAb associated with iodine intake in humans, similarly to what is observed in animals, is likely due to an increased immunogenicity of Tg [30, 34–36]. This may be explained by the fact that Tg is the only self-antigen that undergoes post-translational modification as a consequence of the environmental supply of iodine, with the exposure of previously hidden epitopes. Direct evidence supporting the latter mechanism was recently provided by Latrofa et al. [37] who carried out a detailed study of the fine specificity of TgAb detected in sera of an endemic population before and after implementation of iodine prophylaxis.

However, while the role of the iodine intake level in the etiology of non-autoimmune thyroid disorders is well established, the association between population iodine intake level and AITD is more devious [6]. Taking into account the global health benefit of iodine prophylaxis implementation, the question arises as to whether the implementation of universal salt iodization has led to an increased incidence of AITD and, if so, what is its clinical relevance/impact.

## Does iodoprophylaxis induce thyroid autoimmunity?

As above mentioned, it is well known that variable iodine intake may be accompanied by thyroid disease: adverse consequences of low iodine intake are a major epidemiological problem, but thyroid dysfunction may also occur at higher intake levels [38–40].

In subjects with an underlying thyroid disorder (underlying autoimmune thyroiditis, previous treatment with radioactive iodine or history of external thyroid irradiation, previous subtotal thyroidectomy, postpartum or subacute thyroiditis and intake of some medications, such as lithium, which interferes with iodine organification and thyroid hormones release), acute excessive iodine intakes may lead to temporary overt or subclinical hypothyroidism that resolves when iodine intakes decrease [41].

Moreover, many studies indicate that even relatively small changes in the level of iodine intake of a population may result in changes in the spectrum of thyroid diseases [42]. Iodine-induced hyperthyroidism has been observed at daily iodine intakes of less than 300 µg [43, 44]. An increased incidence of hyperthyroidism in previously iodine-deficient areas has been described, although transiently, even during careful implementation of universal salt iodization [15, 45–47]. Therefore, the World Health Organization (WHO) recommends that iodine fortification of a population should be initiated cautiously and be followed by a monitoring program to register its effects and counteract any side events.

It has been proposed the term “iodine memory” [15] when evaluating the association between current iodine intake and the epidemiology of thyroid disease in a population, because the degree of a previous exposure to different level of iodine intake (low or high) may have influenced the current occurrences of diseases. An increase from low to normal iodine intake is associated with a reduction in thyroid size in the population within a few years [48], while thyroid nodularity is more or less irreversible [49]. It remains to be elucidated if the high frequency of hypothyroidism in areas with excessive iodine intake is a reversible phenomenon. Studies of hypothyroid patients living in Japan and Korea with high iodine intake have indicated that a reduction in iodine intake may normalize thyroid function in some of these patients [50, 51]. Individuals with preexisting thyroid disease or those previously exposed to iodine deficiency may be more susceptible to thyroid disorders due to an increase in iodine intake, in some cases at intakes only slightly above physiological needs. Thyroid dysfunction due to excess iodine intake is usually mild and transient, but iodine-induced hyperthyroidism can be life-threatening in some individuals [21].

The question arises as to such “side pathological effects” are related to the development of thyroid autoimmunity, although there is no doubt that other factors play a role (specifically, the development of hyperthyroidism in iodine-deficient areas where the prevalence of nodular goiter, and consequently the likelihood of nodular functional autonomy, is high).

The effect of public iodization programs on the development of thyroid antibodies is yet uncertain. As previously described, high iodine intake may trigger thyroid autoimmunity, and it might be expected that the prevalence of harboring circulating thyroid antibodies would be low in populations with a low iodine intake and high in populations with a high iodine intake. However, the association between iodine intake and the presence of circulating thyroid antibodies is considerably more complex, taking also into consideration that multiple genetic and environmental factors are involved in the pathogenesis of autoimmune thyroiditis.

The most common autoantibodies against the thyroid gland associated with autoimmune thyroiditis are thyroid peroxidase antibody (TPOAb) and thyroglobulin antibody (TgAb) [52]. Antibody levels seem to correlate well with the severity of histological thyroiditis [53], but in some cases, histological evidence of autoimmune thyroiditis is not accompanied by detectable TPOAb or TgAb in blood [54].

As already noted, it is difficult to interpret and compare the many studies where both thyroid antibodies and iodine status have been measured in one or more populations [55].

Iodine deficiency has been associated with goiter and people with nodular goiter relatively often have circulating thyroid antibodies [56, 57], probably caused by enhanced release of thyroid antigens from the abnormal gland. Thus, circulating thyroid antibodies are probably more common in populations where goiter is common because of iodine deficiency. Consistently with such a scenario, recent findings indicate that iodine deficiency in early pregnancy is associated with higher risk of TPOAb and TgAb positivity and that the presence of both autoantibodies at high titers is associated with increased risk of subclinical or overt hypothyroidism [58], and optimal iodine supply results in the lowest risk for thyroid autoantibody positivity [59].

It has also been reported that the prevalence of lymphocytic thyroid infiltration becomes high after an increase in population iodine intake [60]. Moreover, a sudden increase in iodine intake in an iodine-deficient population may induce enhanced thyroid autoimmunity [61], but this may be a transient phenomenon [62].

Two groups of Sri Lankan schoolgirls were studied in 1998 and 2001 in relation to the evolution of thyroid autoimmunity, with the change in goiter prevalence, during 3 years of iodine prophylaxis [63]. This study demonstrates that in 2001, goiter prevalence and thyroid autoimmunity rates were significantly lower than in 1998; the pattern of thyroid Ab was different in the two groups (more TPOAb in 2001 group); in 2001 alone, the occurrence of hypothyroidism was correlated with the presence of thyroid autoimmunity. These results indicated, for the first time, an evolution of thyroid autoimmunity markers during the course of iodine prophylaxis: when iodine supplementation is first introduced it may have induced the greater prevalence of TgAb in 1998 in comparison with 2001. It is noteworthy that the reversibility of thyroid autoimmunity occurred mainly in those individuals who showed low titers of TgAb alone in 1998: this fact suggests that the immune system’s response to iodine intake could be heterogeneous, possibly reflecting different immunological background.

In Greece, thyroid autoimmunity has been positively associated with increased iodine intake, as well as with the female gender [64]. Moreover, mean TSH values were increased in females and decreased with age. The latter is probably due to the presence of autonomous goiter in older subjects, as a result of long-term status of iodine deficiency in the past.

As well, an increased prevalence of thyroid autoantibody positivity and subclinical hypothyroidism was observed in Denmark, after 4–5 years from the implementation of a cautious iodoprophylaxis program in 3570 members from the original cohort [65]. Similar results were obtained in the Pescopagano’s survey, a small rural community followed up for as long as 15 years from iodoprophylaxis with iodized salt implementation, which in turn resulted in marked reduction of the prevalence of goiter and thyroid autonomy [66].

**Fig. 1:**
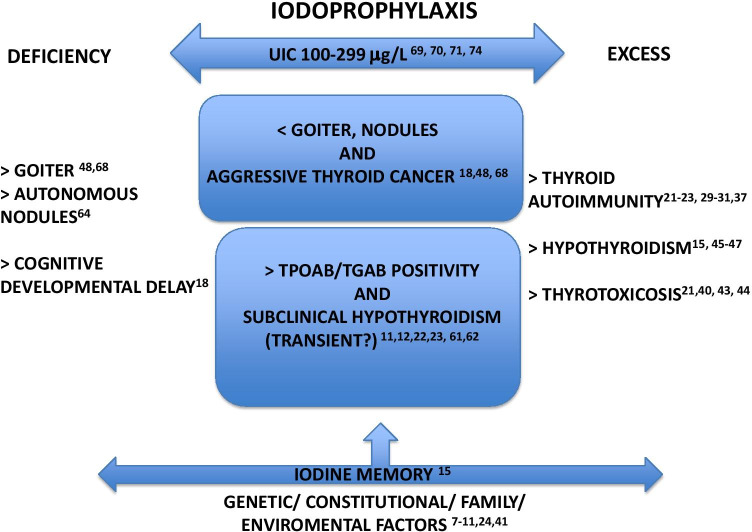
Summary of expected effects of iodine deficiency/excess and iodoprophylaxis. Reference numbers are the same as in the main text. UIC: urinary iodine concentration

However, other studies report no increase or a reduction in thyroid autoimmunity with time following iodine salt fortification. A large (more than 350,000 people) retrospective study conducted in Tasmania between 1995 (median population UIC: 75 μg/L) and 2013 (median population UIC: 108 μg/L) and aimed at analyzing the relationship between trends in TSH and TPOAb testing and different phases of iodine nutrition failed to detect any increasing trend over time of TPOAb following iodine salt fortification, whereas a decreasing trend of overt thyroid dysfunction was apparent [67]. Another study, which analyzed the impact of iodine nutrition on thyroid diseases few years after the introduction of the iodine prophylaxis program (1997–2001, median population UIC: 123 μg/L) and more recently in Northeast Germany (2008–2012, median population UIC: 112 μg/L), also found a stable prevalence of markers of thyroid autoimmune disorders and a decreased prevalence of goiter [68]. More recently, in a study conducted in China after two decades of universal salt iodination program, a stable prevalence of thyroid antibody positivity in the population has been reported. This study also found an inverse relationship between iodine intake and thyroid antibodies, suggesting that UIC between 100 and 300 μg/L is optimal and safe for thyroid autoimmunity [69]. More specifically, a number of data suggest that only iodine supplementation regimens attaining relatively high median UIC levels (i.e., 200 µg/L or more) are able to induce relevant long-term alteration of thyroid function due to autoimmune disease, and the relationship between population iodine supply and occurrence of AITD could be depicted by a “U-shaped” function [70, 71]: in other words, the likelihood of developing thyroid autoimmune diseases would increase when median population UIC exceeds approximately 300 µg/L, but as well when is less than 100 µg/L (see Fig. 1). The majority of these results come from Chinese populations, in whom marked differences in iodine supply in single region have been observed due to different iodine supply strategies [69, 71–75]. In line with such a scenario, according to at least two recent reports cited above [58, 59] both iodine deficiency and iodine excess are associated with increased risk of thyroid autoantibody positivity in pregnancy.

Moreover, circulating thyroid antibodies are not extraordinarily common in populations with stable high iodine intake [38], although thyroid dysfunction is more frequent when the iodine intake is high [76].

Importantly, most recent data from the Danish survey in North Jutland, involving an open cohort of over 300,000 individuals, demonstrated no increase of overt hypothyroidism (related, as a rule, to autoimmune thyroiditis) and a decrease of hyperthyroidism (due to thyroid autonomy, but also due to autoimmunity, i.e., Graves’ disease) over a period of more than 15 years following implementation of mandatory iodine fortification [77].

Finally, and on the same line of evidence, the last data derived from the Sri Lanka iodoprophylaxis survey showed that more than 20 years after its implementation the prevalence of TgAb consistently declined, and no increase of the prevalence of hypothyroidism (even subclinical) was recorded [78].

Thus, although several reports suggest a somehow promoting effect of iodine salt fortification on thyroid autoimmunity, its role in causing clinically relevant autoimmune thyroid disease in long term is not clearly proven.

### Discussion

As described above, the epidemiology of thyroid diseases has undergone profound changes since the implementation of iodoprophylaxis, specifically resulting in decrease of the prevalence of goiter and neonatal hypothyroidism, as well as in improved cognitive function development in infancy and reduced incidence of more aggressive forms of thyroid neoplasia. Thus, there is no doubt about the benefits of iodoprophylaxis for the general population and specifically for newborns, children, and pregnant women. The main question we have intended to address with the present review is the following: is an increase of thyroid autoimmunity a price to pay for such benefits, or the risk of a clinically significant increase of autoimmune disease is neglectable?

Undoubtedly, it is quite difficult to provide a definite evidence-based answer to the main question (does iodoprophylaxis increase the prevalence of clinically significant thyroid dysfunction due to thyroid autoimmunity?) for a number of reasons:

- Definition of clinically apparent thyroid autoimmune disease or simply autoantibody prevalence: as generally recognized, autoantibody positivity, especially for TPOAb, confers a risk of, but not necessarily results in progression to clinical disease.
- Possible transient effects: autoantibody positivity, especially at low titers, and subclinical hypothyroidism (i.e., slight increase of TSH with Thyroxine concentrations within the normal range) may be transient.
- Possible effect of iodine status (mild or moderate/severe iodine deficiency) before iodoprophylaxis implementation.
- Evolution of autoimmune disease epidemiology independently of iodine supply.

As far as the first two points are concerned, it has to be noted that the numerous published studies, cited in the previous section, are quite heterogeneous, so that a meta-analysis, or also a rigorous systematic review, is hardly feasible. Salt iodization has been implemented and promoted in different ways, either mandatory or on a voluntary basis, in different ethnic groups, at different levels of baseline iodine intake. Most studies are cross-sectional, in some cases evaluating individuals of the same ethnic group living in areas with different iodine intake, or were repeated years (up to two decades) after prophylaxis implementation: numbers of evaluated individuals ranged from hundreds to tens/hundreds of thousands.

In addition, TPOAb and thyroid function have been evaluated in the majority of studies. As described above, an increase of thyroid autoantibodies (TPOAb and/or TgAb) following implementation of prophylaxis by means of iodized salt, and an increased prevalence of subclinical hypothyroidism, has been consistently reported. However, in a significant proportion of patients, subclinical hypothyroidism may reverse to full euthyroidism [79]. Again, it is worth noting that in a number of studies, no significant increase of thyroid autoimmunity was observed when iodine intake was adequate.

Most often, published studies refer to autoantibody (especially TPOAb) positivity as an indicator of the presence of, or risk to develop thyroid autoimmune disease, with consequent thyroid dysfunction (more often, subclinical hypothyroidism). However, a reliable indicator of structural thyroid damage due to autoimmune thyroiditis is thyroid ultrasonography, whose specificity and sensitivity under this respect have been underlined [80, 81]. Only few data, to our knowledge, have been published concerning the prevalence of hypoechoic pattern, typical of autoimmune thyroiditis following implementation of iodoprophylaxis with iodized salt: Miranda et al., in Brazil, reported an increase of hypoechoic pattern in children following attainment of high (300 µg/L or more) UIC concentration, and a trend to decrease when the target was adjusted at 165 µg/L [73]. In addition, in a recent study reporting data after a decade of iodine prophylaxis on voluntary basis in Italy [82], the frequency of moderately or markedly hypoechoic patterns in schoolchildren residing in iodine-sufficient areas (median UIC value 129 μg/L) was 6.6%, whereas it was significantly higher (10.9%) in an area of Sicily region where UIC value was still under the desired target value (median UIC value 89 μg/L). Even more importantly, in Liguria, one of the studied Italian regions where comparative dataset collected 8 years before (i.e., 2 years following iodized salt consumption implementation) were available, no increase in the frequency of hypoechoic pattern was observed (3.3% at 2, 2.7% at 10 years) despite a significant reduction in goiter prevalence. These data suggest that iodoprophylaxis aimed to attain an UIC target indicative of an adequate iodine intake does not result in a structural damage of the thyroid typical of autoimmune thyroiditis.

Concerning the third point, it has been suggested that the possible inducing effect of iodine supply on thyroid autoimmunity is at least in part related to baseline iodine status, namely, the higher the degree of iodine deficiency, the higher the likelihood of autoimmunity induction.

That iodine supply in conditions of severe iodine deficiency may cause thyroid dysfunction is a well-known fact, but it is mainly related to the development of hyperthyroidism due to thyroid autonomy in nodular goiters [43]: nowadays, however, due to the present iodine nutritional status in the majority of countries [19], the risk of toxicity of autonomously functioning thyroid nodules induced by sudden iodine repletion is probably minimal. On the other hand, data on the relationship between baseline iodine status and autoimmunity development are at least in part contradictory. In any case, considering the global amelioration of iodine nutrition worldwide and the reduction of impact of moderate/severe iodine deficiency, the relevance of this point is probably minor.

Concerning the fourth point, namely, the general evolution of autoimmune disease epidemiology, there is little doubt that the prevalence of autoimmune diseases has progressively increased during the last decades [83, 84], with an inverse correlation with infectious disease epidemiology and a direct correlation with “well-being” indicators (income, improved hygienic conditions), in both developed and developing countries [85]. More than 10 years ago, proof of such a relationship was specifically provided for thyroid autoimmunity [86]. This implies that studies evaluating the increase in thyroid autoimmune diseases following iodoprophylaxis implementation over a decade or more may be affected by the bias of the general trend to increase in autoimmune phenomena per se. Such a trend is hardly quantifiable but also may be hardly ruled out.

In summary, literature data are at least in part controversial, as previously reported in detail. However, on some points, there is substantial agreement:

- Iodine excess (UIC > 300 µg/L) (see Table 2) may result in altered thyroid function, and this is at least in part due to the development of thyroid autoimmunity. The molecular mechanisms whereby iodine affects the development of thyroid autoimmunity are known or conceivable.
- Implementation of a not adequately monitored iodine prophylaxis may result in increased evidence of “autoimmune phenomena” concerning the thyroid gland.
- The clinical impact of such phenomena (either transient or evolving in clinically apparent thyroid dysfunction) mainly depends upon the target level of iodine supply attained.

**Table 2:** Maximum recommended daily intake levels of iodine and corresponding upper urinary iodine concentration (UIC) thresholds for iodine excess ( modified from ref [21])

	IOM(µg/die)	SCF (µg/die)	WHO(µg/kg/die)	UIC (µg/L)
7–10 yr	600 (9–13 aa)	300	50 (7–12 aa)	300
Adults	1100	600		ND
Pregnant & lactating women	1100	600	40	500

*IOM*, United States Institute of Medicine; *SCF*, The European Union Scientific Committee on Foods; *WHO*, World Health Organization; *UIC*, urinary iodine concentration; *ND*, not determined

As a matter of fact, consistent data support the concept that implementation of iodoprophylaxis programs aimed to reach iodine sufficiency and at the same time to avoid iodine excess (i.e., maintaining population UIC within a relatively narrow interval, as in many European countries, including Italy) does not definitely result in long-term increased incidence of clinically relevant autoimmune thyroid disease (see Fig. 1). Under this respect, the last data from Danish and Sri Lanka surveys showing no increase of hypothyroidism incidence and a decrease of hyperthyroidism due to Graves’ disease about two decades after starting iodoprophylaxis program are of particular interest, as well as the results of recent studies on Chinese populations [69, 74, 77]. As mentioned above, a “U-shaped” relationship between iodine intake and the risk of developing thyroid autoimmune disease is conceivable, namely, both iodine excess (even moderate) and also iodine deficiency may increase such risk [71].

### Conclusion

We can conclude that an increased incidence of “autoimmune thyroid phenomena” following implementation of iodoprophylaxis by means of iodine-enriched salt consumption on a voluntary basis is likely; however, its long-term clinical relevance is probably scarce. Nonetheless, a population iodine supply higher than adequate due to a poorly controlled iodoprophylaxis could induce clinically relevant thyroid dysfunction. Therefore, monitoring iodoprophylaxis with standardized population surveys is mandatory, to ensure that both iodine deficiency and iodine excess are avoided. In this regard and on the basis of the experience from Italy and other countries [20, 69], we are confident that the routine assessment of thyroid hypoechogenicity in monitoring iodine prophylaxis programs in populations will provide the advantage of easily and quickly identifying even minimal modifications of this frequency over time and to allow the appropriate adjustments of the iodine prophylaxis program if necessary.

Finally, since studies showing a lack of increased thyroid autoimmunity after implementation of universal salt iodization program have reported median UIC values ranging 100–199 μg/L if conducted in children [62] and 100–299 μg/L if conducted in adults [70], we acknowledge the need for further studies aimed at evaluating the possible advantage of using narrow age-related UIC intervals upon which iodine sufficiency is achieved and increase in the baseline frequency of thyroid autoimmunity is avoided at all classes of age.

## Abbreviations

AITD: Autoimmune thyroid diseases
AT: Autoimmune thyroiditis
GD: Graves’ disease
TPOAb: Thyroid peroxidase antibodies
TgAb: Thyroglobulin antibodies
Tg: Thyroglobulin
TRAb: TSH-receptor autoantibodies
IDD: Iodine deficiency disorders
IQ: Intelligence quotient
USI: Universal salt iodization
WHO: World Health Organization
UIC: Urinary iodine concentration
IOM: United States Institute of Medicine
EAR: Estimated average requirement
AI: Adequate intake
RDA: Recommended daily allowance
RNI: Recommended nutrient intake
SCF: The European Union Scientific Committee on Foods
ND: Not determined
